# NOTCH Receptors and DLK Proteins Enhance Brown Adipogenesis in Mesenchymal C3H10T1/2 Cells

**DOI:** 10.3390/cells9092032

**Published:** 2020-09-04

**Authors:** María-Milagros Rodríguez-Cano, María-Julia González-Gómez, Beatriz Sánchez-Solana, Eva-María Monsalve, María-José M. Díaz-Guerra, Jorge Laborda, María-Luisa Nueda, Victoriano Baladrón

**Affiliations:** 1 Departamento de Química Inorgánica, Laboratorio de Bioquímica y Biología Molecular, Facultad de Farmacia/CRIB/Unidad de Biomedicina, Orgánica y Bioquímica, Universidad de Castilla-La Mancha/CSIC, C/Almansa 14, 02008 Albacete, Spain; Maria.RodriguezCano@uclm.es (M.-M.R.-C.); MariaJulia.Gonzalez@uclm.es (M.-J.G.-G.); 2 National Institutes of Health, Laboratory of Cellular Oncology, Center for Cancer Research, National Cancer Institute, Bethesda, MD 20892, USA; beatriz.sanchez-solana@nih.gov; 3 Departamento de Química Inorgánica, Laboratorio de Bioquímica y Biología Molecular, Facultad de Medicina de Albacete/CRIB/Unidad de Biomedicina, Orgánica y Bioquímica, Universidad de Castilla-La Mancha/CSIC, C/Almansa 14, 02008 Albacete, Spain; EvaMaria.Monsalve@uclm.es (E.-M.M.); MariaJose.Martinez@uclm.es (M.-J.M.D.-G.)

**Keywords:** EGF-like proteins, mesenchymal cells, preadipocytes, adipogenic differentiation, brown-like adipocytes

## Abstract

The NOTCH family of receptors and ligands is involved in numerous cell differentiation processes, including adipogenesis. We recently showed that overexpression of each of the four NOTCH receptors in 3T3-L1 preadipocytes enhances adipogenesis and modulates the acquisition of the mature adipocyte phenotype. We also revealed that DLK proteins modulate the adipogenesis of 3T3-L1 preadipocytes and mesenchymal C3H10T1/2 cells in an opposite way, despite their function as non-canonical inhibitory ligands of NOTCH receptors. In this work, we used multipotent C3H10T1/2 cells as an adipogenic model. We used standard adipogenic procedures and analyzed different parameters by using quantitative-polymerase chain reaction (qPCR), quantitative reverse transcription-polymerase chain reaction (qRT-PCR), luciferase, Western blot, and metabolic assays. We revealed that C3H10T1/2 multipotent cells show higher levels of NOTCH receptors expression and activity and lower *Dlk* gene expression levels than 3T3-L1 preadipocytes. We found that the overexpression of NOTCH receptors enhanced C3H10T1/2 adipogenesis levels, and the overexpression of NOTCH receptors and DLK (DELTA-like homolog) proteins modulated the conversion of cells towards a brown-like adipocyte phenotype. These and our prior results with 3T3-L1 preadipocytes strengthen the idea that, depending on the cellular context, a precise and highly regulated level of global NOTCH signaling is necessary to allow adipogenesis and determine the mature adipocyte phenotype.

## 1. Introduction

The NOTCH signaling pathway is vitally involved in most cell differentiation and proliferation processes during both developmental and adult stages [1,2,3]. Mammals possess four NOTCH receptors and five canonical ligands (JAG (JAGGED canonical NOTCH ligand) 1 and 2, and DLL (DELTA-Like Canonical NOTCH Ligand) 1, 3, and 4) [4,5,6]. The NOTCH family of receptors and ligands also includes non-canonical transmembrane ligands that inhibit NOTCH signaling, such as DLK1 (DELTA-like 1 homolog) protein (Delta-like 1 homolog), DLK2 (DELTA-like 2 homolog) protein (Delta-like 2 homolog) [7,8], EGFL7 protein (Epidermal growth factor-like protein 7) [9], and DNER protein (DELTA/NOTCH EGF-like Repeat Containing) [10].

Activation of NOTCH receptors ensues after their interaction with a canonical ligand [11,12,13,14]. After this interaction, proteolytic events release the intracellular active domain of the NOTCH receptors (NICD), which is then relocated to the nucleus where it binds to CSL/RBPJκ (CBF1, Suppressor of Hairless, Lag-1/recombination signal binding protein for immunoglobulin kappa J region) factor and other co-activators to modulate the expression of HES (hairy and enhancer of split)/HEY (hairy and enhancer-of-split related with YRPW motif) transcription factors [14,15,16].

The molecular partners of DLK proteins and their mechanisms of action are not completely elucidated yet. We were the first to reveal that both DLK proteins interact with the four NOTCH receptors and function as inhibitory non-canonical ligands of NOTCH signaling in a dose-dependent manner [8,17,18,19,20,21,22,23]. DLK1 and DLK2 proteins lack a DSL (DELTA-SERRATE-LAG-2) domain, which is required for interactions between NOTCH receptors and their canonical ligands [12,14,24]. However, both proteins possess conserved DOS (DELTA and OSM-11) domains thought to be involved in NOTCH/DSL activation in competition with NOTCH canonical ligands [4,25].

The NOTCH signaling pathway modulates a complex network of other signaling pathways that participate in the conversion of preadipocytes or mesenchymal stem cells into mature adipocytes [26,27]. However, the role of NOTCH signaling in adipogenesis is highly controversial. Some authors claim it to be dispensable for adipocyte differentiation [28], whereas others have shown positive or negative roles of NOTCH signaling in the adipogenesis process [18,19,29,30,31,32,33,34,35]. Accumulated evidence indicates that the DLK1 protein is involved in adipogenesis. Numerous works, some of them performed with 3T3-L1 preadipocytes and *Dlk1-*deficient or *Dlk1*-transgenic mice, point to *Dlk1* gene as a positive or negative modulator of adipogenesis, depending on the cellular context. *Dlk1* gene is also involved in the development of obesity and diabetes [18,19,35,36,37,38,39,40,41,42,43,44,45]. Furthermore, Dlk2 gene also modulates adipogenesis in 3T3-L1 and C3H10T1/2 cells [8,46].

The existence of two types of adipose tissue is well known. White adipose tissue (WAT) is responsible for lipid and energy storage. Furthermore, WAT not only contains adipocytes but also a wide population of other cells, including immune cells, mesenchymal stem cells (MSCs), and adipose precursor cells. On the other hand, brown adipose tissue (BAT) has been found in rodents, hibernating mammals, and humans [26,47,48,49,50,51]. In humans, WAT also contains some brown adipocytes and adaptive thermogenic beige adipocytes [52,53,54,55,56,57,58,59]. Finally, BAT and beige adipocytes have been shown to be endocrine/autocrine organs.

Brown adipocytes promote energy expenditure for thermogenesis via the mitochondrial uncoupling protein 1 (UCP-1), which is involved in the last steps of the thermogenic program [26,47,48,49,50,51,60,61,62,63,64,65]. Brown adipocytes express many other brown-fat signatures, such as the transcription co-regulator PR domain-containing 16 (PRMD16) [26,66] and the peroxisome proliferator activated receptor gamma coactivator 1-alpha (PGC1α) [67]. *Gyk*, which encodes for a glycerol kinase activated in brown adipocytes [68,69]; CIDEA (cell death-inducing DNA fragmentation factor, alpha subunit-like effector A), a highly expressed protein in lipid droplet membranes and mitochondria of brown adipocytes [70]; and *Sirt1* (Sirtuin 1), which is involved in the promotion of the thermogenic program [71,72,73], are three other markers characteristic of brown adipogenesis and mitochondrial biogenesis.

Some publications have indicated that NOTCH signaling regulates energy metabolism and modulates the mature adipose phenotype by inhibiting or activating the brown phenotype conversion [74,75,76]. However, other studies have demonstrated that blocking NOTCH signaling in post-development adipocytes has no effect on systemic glucose and lipid metabolism [77]. Recent studies also implicate *Dlk*1 gene in the control of whole-body metabolism [78,79], the onset of diabetes in humans and mice [45,80,81], and adipocyte browning [18,19,82,83]. Recently, we demonstrated that *Notch1* gene could be involved in the conversion of 3T3-L1 preadipocytes to a brown-like phenotype, whereas the rest of the *Notch* and *Dlk* genes lead 3T3-L1 preadipocytes towards the acquisition of a white adipocyte phenotype [18,19]. The results obtained in this work suggest that a precise level of global NOTCH signaling, which may depend on the cellular context, is necessary to allow the adipogenesis process of multipotent C3H10T1/2 cells and to reach a given mature adipocyte phenotype, as shown in 3T3-L1 preadipocytes.

## 2. Materials and Methods

### 2.1. Plasmids, Cell Culture, and Transfections

Transformation of *Escherichia coli* TOP10 competent cells and plasmid DNA isolation and purification were performed as previously described [8,35]. Plasmids pCDLK1 (DLK1) and pCDLK2 (DLK2) contain the complete cDNA sequence of the *Dlk1* gene and the *Dlk2* gene in sense orientation, respectively [8,35]. Plasmid pC-N1 (N1S) contains the complete mouse *Notch1* gene cDNA (ATCC clone: MBA-105) in sense orientation [22]. Plasmid pCN-N2 (N2S) contains the complete mouse *Notch2* gene cDNA [18,19]. Plasmid pEntry-N3 (N3S) contains the complete mouse *Notch3* gene cDNA sequence [18,19]. Plasmid pGF-N4 (N4S) contains the complete mouse *Notch4* gene cDNA sequence [18,19]. Plasmids pN-HES1 (H1S) and pN-JAG1 (JAG1S) drive the expression of the complete HES1 (hairy and enhancer of split 1) and JAG1 (JAGGED canonical NOTCH ligand 1) proteins, respectively [22,84]. Plasmids pL-DLK1e (sDLK1) and pL-DLK2e (sDLK2) contain the cDNAs encoding for the extracellular soluble regions of the DLK1 and DLK2 proteins, respectively [22,84]. Plasmids pL-JAG1e and pL-DLL4e contain the cDNA encoding for the extracellular soluble regions of JAG1 (sJAG1) and DELTA4 (DELTA-Like Canonical NOTCH Ligand 4) (sDLL4), respectively [22,84]. Finally, plasmid pNICD1 contains 2500 bp encoding for the intracellular domain of the NOTCH1 receptor [18,19].

Mesenchymal C3H10T1/2 cells (C3H; ATCC CCL-226, clone 8), 3T3-L1 preadipocytes (L1; ATCC CCL-92.1), and HEK 293T/17 (ATCC CRL-11268) cells were used. Pools of C3H10T1/2 cells stably or transiently transfected with *Notch* or *Dlk* genes expression plasmids were employed. HEK 293T/17 were transiently transfected with plasmids expressing the soluble forms of DLK1, DLK2, JAG1, or DLL4 proteins. Cell culture procedures were performed as previously described [8,18,19,22]. Transfections were performed in 50% confluent cells cultured in six-well plates using 1 microgram of plasmid and Superfect reagent (Qiagen), following the procedures recommended by the company. Transiently transfected cells were analyzed 48 h after transfection. Stably transfected cells were selected after treatment with the appropriated concentration of G418 antibiotic.

### 2.2. Quantitative PCR and RT-PCR mRNA Transcription Analysis

Confluent cell monolayers of stably or transiently transfected cells were processed to obtain total RNA and cDNA, which were used to perform qRT-PCR gene expression assays [22]. Total RNA was isolated by using the RNeasy Kit (Qiagen). RNA concentration and purity (260 nm/280 nm absorbance ratio) was analyzed using a NanoDrop One spectrophotometer (Thermo Scientific). cDNAs were obtained from 1 microgram of total RNA using a cDNA synthesis kit (Fermentas). To perform gene expression assays, total cDNAs were used as templates in quantitative RT-PCRs with the StepOne Plus qRT-PCR system (Applied Biosystems), using Fast SYBR Green Master Mix. The primers used to determine *Dlk, Notch, Hey1,* and *Hes1* genes expression levels were described previously [8,17,18,19]. The primers used to analyze the expression levels of adipogenic and mitochondrial biogenesis markers are described in Section 2.6. *P0* mRNA transcription was used as a control to compare the C_T_ (cycle threshold) values from the different samples in all qPCR and qRT-PCR experiments. P0 riboprotein [85] is a constitutively expressed protein, the expression of which does not change with adipogenic treatment or when the studied genes are overexpressed. Expression analyses were repeated at least three times.

### 2.3. Culture Supernatants and Conditioned Media

HEK 293T/17 cells were transiently transfected with empty-vector or the plasmids pL-DLK1 (sDLK1), pL-DLK2 (sDLK2), pL-JAG1 (sJAG1), and pL-DLL4 (sDLL4), which overexpressed the respective soluble forms of the proteins. Secretion of these proteins to the culture medium was analyzed by Western blotting after filtration and concentration of supernatants with centricons (Millipore). These culture media were used as conditioned culture media in adipogenic assays, as previously described [22,84].

### 2.4. Protein Sample Preparation and Western Blotting

Protein extracts from cultured cells or cell culture supernatants were quantified and electrophoresed as previously described [22]. Western blotting was performed using the appropriated dilutions of primary and secondary antibodies (usually 1:2000) (Table 1). Detection of α-tubulin with a specific antibody (Sigma) was used as a protein-loading control.

Colorimetric determinations to quantify total protein amounts in cell extracts and culture supernatants were performed with a Plate Reader Axis UVM340 (Biochrom). Western blotting images were obtained by developing exposed films (CP-BU New, Agfa) for 10–30 s (α-tubulin and HA fusion proteins), 1 min (DLK1, DLK2, NOTCH1, and NOTCH2 proteins), or 5 min (NOTCH3 and NOTCH4 proteins) with the Pierce ECL Plus Western blotting substrate kit (Thermo Scientific) in a Curix 60 developing apparatus (AGFA). Films were scanned with an HP Officejet Pro 8600 scanner and signals of the different proteins were quantified using the QuantityOne 4.6.5. (Basic) software.

### 2.5. Luciferase Assays

NOTCH transcriptional activity was analyzed using luciferase assays, as previously described [18,19,22,84]. We also treated C3H10T1/2 cells with the γ-secretase inhibitor DAPT (N-[N-(3,5-Difluorophenacetyl)-L-alanyl]-S-phenylglycine t-butyl ester) (10 μM), used as a NOTCH-signaling-inhibition control. Luciferase assays were measured using the Orion II microplate luminometer (Berthold), and samples were processed with the Dual-Luciferase Reporter Assay System (Promega), following the supplier’s recommendations. Assays were repeated at least three times.

### 2.6. Adipogenic Assays and Mitochondrial Biogenesis Analysis

Induction of C3H10T1/2 adipogenesis and adipocyte staining with oil red O were performed according to standard procedures, as previously described [8,35]. When appropriate, cells were induced to differentiate in the presence of control-conditioned media (CM) or conditioned media containing sDLK1, sDLK2, sJAG1, or sDLL4 soluble proteins throughout the entire differentiation process. Microscopic images were visualized through different objectives of a Motic AE31 microscope connected to a Moticam 2300 camera (3.0 MPixel USB 2.0). Cell images were acquired with the software Motic Images Plus 2.0, using the standard parameters (exposition: 418.9, gamma: 0.8019). Assays were repeated at least three times.

We determined adipocyte differentiation levels by analyzing the expression of the early, medium, and late adipocyte differentiation markers *Cebpb* (CCAAT Enhancer Binding Protein Beta)*, Pparg* (peroxisome proliferator activated receptor gamma), and *aP2* (adipocyte protein 2/fatty acid binding protein 4), respectively, as previously described [86], seven days after induction of adipogenesis. We also measured *Ucp2* (uncoupling protein 2) marker expression, which presents a higher expression levels in white adipose tissue. To further study the phenotype of terminally differentiated C3H10T1/2 adipocytes, we also analyzed the expression of the brown adipose and mitochondrial biogenesis markers *Prdm16* (PR domain-containing 16), *Pgc1a* (peroxisome proliferator activated receptor gamma coactivator 1-alpha)*, Ucp1* (mitochondrial uncoupling protein 1), *Gyk*, *Cidea,* and *Sirt1,* as previously described [18,19].

Finally, we estimated mitochondrial biogenesis by determining the DNA levels of the mitochondrial *CytB* gene via qPCR analysis and normalizing them to the DNA levels of the nuclear *ApoB* (apoliprotein B) gene in terminally differentiated cells. The oligonucleotides used for this analysis were described previously [18,19,87,88].

### 2.7. Lipolytic Potential, Lactate Release to the Extracellular Medium, and Oxygen Consumption Rate (OCR) Assays

The amounts of glycerol and extracellular lactate released to the culture medium were determined using lipolysis and lactate colorimetric assay kits (BioVision), respectively. To measure OCR, we used the oxygen consumption rate assay kit (Abcam). In these assays, we plated 15,000 cells per well in 96-well plates, proceeded with the adipogenic differentiation protocol, and, finally, performed the assays following the manufacturers’ recommendations. Colorimetric determinations to quantify extracellular lactate in the culture medium and the release of glycerol to the medium were performed with a Plate Reader Axis UVM340 (Biochrom). To measure the OCRs of the different cultured adipocytes, we used an F-7000 fluorescence spectrophotometer (Hitachi). Data were normalized to the amount of total protein in each well.

### 2.8. Statistical Analysis

The fold activation or inhibition in each sample was calculated and normalized relative to control sample, which was set arbitrarily at 1. Data are represented as the mean ± SD of at least three different independent experiments performed in triplicate. Data were analyzed with Student’s *t*-test to determine statistical significance. A *p*-value ≤ 0.05 was considered statistically significant (*); a *p*-value ≤ 0.01 was considered highly statistically significant (**); a *p*-value ≤ 0.001 was considered extremely statistically significant (***). Statistical non-significance is indicated by ns.

## 3. Results

### 3.1. Comparison of Adipogenesis Levels and Expression Levels of Some of the Notch Family Genes between Multipotent C3H10T1/2 Cells and 3T3-L1 Preadipocytes

The size of the fat droplets in C3H10T1/2 and 3T3-L1 adipocytes was similar and was more typical of a brown adipocyte phenotype than of a white one (Supplementary Figure S1A, 400× magnification images). However, each cell line showed a different basal level of adipogenesis, being higher in 3T3-L1 preadipocytes than in C3H10T1/2 multipotent cells (Supplementary Figure S1A, 100× magnification images). These differences were probably due to their distinct differentiation status, since 3T3-L1 cells are already committed to adipocyte differentiation, but C3H10T1/2 cells can differentiate into different terminal phenotypes besides adipocytes.

We first determined using qRT-PCR the relative mRNA transcription levels of *Dlk* and *Notch* genes, and two NOTCH receptors’ target genes (*Hes1* and *Hey1*) in non-differentiated C3H10T1/2 cells. The highest levels corresponded to *Notch1*, *Notch2*, *Hes1,* and *Dlk1* genes (Supplementary Figure S1B). We then compared the basal expression levels of the four *Notch* genes, some of the NOTCH receptors’ target genes, and the two *Dlk* genes between non-differentiated C3H10T1/2 and 3T3-L1 cells. Quantitative RT-PCR assays showed that multipotent C3H10T1/2 cells displayed lower *Dlk* mRNA expression levels, especially in the case of *Dlk1* gene, than 3T3-L1 preadipocytes (Supplementary Figure S1C). Nevertheless, C3H10T1/2 cells had higher *Notch* mRNA expression levels than 3T3-L1 preadipocytes, especially of *Notch1* and *Notch2* genes (Supplementary Figure S1D). As expected, multipotent C3H10T1/2 cells also presented greater *Hes1* and *Hey1* mRNAs expression levels than 3T3-L1 preadipocytes (Supplementary Figure S1D).

Therefore, these data and other previously published data [35] indicate that C3H10T1/2 cells, used as the adipogenic model in this work, show greater global *Notch* genes expression and NOTCH signaling levels than 3T3-L1 cells, which could be related to their different basal adipogenesis levels in response to adipogenic inductors.

### 3.2. Analysis of the Expression of NOTCH Receptors and DLK Proteins in Multipotent C3H10T1/2 Cells

**Figure 1:**
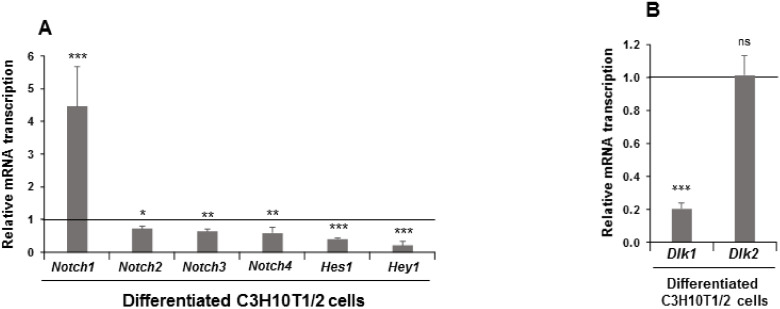
Expression of *Notch* family genes in differentiated C3H10T1/2 cells. Relative mRNA transcription levels of *Notch*, *Hes1* (hairy and enhancer of split 1), and *Hey1* (hairy and enhancer-of-split related with YRPW motif 1) (**A**) and *Dlk* (DELTA-like homologs) (**B**) genes in seven-day differentiated C3H10T1/2 cells. Data from qRT-PCR (quantitative reverse transcription-polymerase chain reaction) and qPCR (quantitative polymerase chain reaction) assays were normalized to *P0* (ribosomal protein P0) mRNA transcription levels. The fold activation or inhibition was calculated relative to levels is non-differentiated C3H10T1/2 cells, which were set arbitrarily at 1 (horizontal black line). Data are shown as the mean ± SD of at least three biological assays performed in triplicate. The statistical significance calculated by Student’s *t*-test is indicated (* *p* ≤ 0.05, ** *p* ≤ 0.01, *** *p* ≤ 0.001). Non-statistical significance is indicated by ns.

We first measured the relative mRNA transcription levels of *Notch* and *Dlk* genes, and of the NOTCH receptors’ target genes *Hes1* and *Hey1*, in C3H10T1/2 cells induced to differentiate to adipocytes (Figure 1).

The expression level at the end of the adipogenic process was downregulated in differentiated cells for all these genes except for *Notch1* gene, for which the expression increased (Figure 1A). As expected, *Dlk1* gene expression diminished with adipogenic treatment, but no significant changes were observed in *Dlk2* gene expression (Figure 1B). To further investigate these initial observations, we decided to stably transfect C3H10T1/2 cells with plasmids that overexpressed each of the four NOTCH receptors to study their effects on adipogenesis outcome and on adipocyte fate. We also generated new stably *Dlk1-* and *Dlk2*-transfected pools to confirm their already established positive effects on C3H10T1/2 adipogenesis [35] and to analyze their role in C3H10T1/2 adipocyte fate. We confirmed using qRT-PCR and Western blotting that these genes were indeed overexpressed. Each of the four stably *Notch-*transfected pools overexpressed the corresponding *Notch* mRNA and protein as compared to its control (Supplementary Figure S2A–D). In the case of stably *Notch1-* and *Notch3-*transfected pools, we detected both the complete protein and the intracellular regions NICD1 and NICD3, respectively (Supplementary Figure S2A,C). However, in the case of stably *Notch2-* or *Notch4-*transfected pools, we only detected either the complete or the intracellular protein, respectively (Supplementary Figure S2B,D).

**Figure 2:**
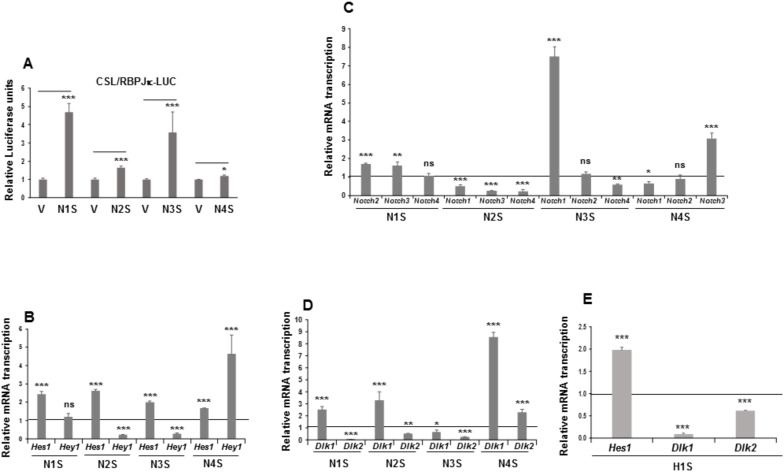
NOTCH signaling in mesenchymal C3H10T1/2 cells stably overexpressing NOTCH receptors or the HES1 protein. (**A**) NOTCH receptors’ transcriptional activity, as measured by luciferase assays, in each of the four stably *Notch*-transfected pools. The relative luciferase activities were always normalized to renilla levels. (**B**) qRT-PCR analysis of the relative *Hes1* and *Hey1* mRNA transcription levels in each of the stably *Notch*-transfected pools. (**C**) qRT-PCR analysis of relative mRNA transcription levels of *Notch* genes in the stable *Notch1* transfectant (N1S), the stable *Notch2* transfectant (N2S), the stable *Notch3* transfectant (N3S), and the stable *Notch4* transfectant (N4S). (**D**) qRT-PCR analysis of *Dlk1* (DELTA-like 1 homolog) and *Dlk2* (DELTA-like 2 homolog) mRNA transcription levels in the stable *Notch* transfectants. (**E**) qRT-PCR analysis of the relative *Hes1* and *Dlk* mRNA transcription levels in the stable *Hes1* gene transfectant (H1S). All these assays were performed using non-differentiated cells. Data in qRT-PCR assays were normalized to *P0* mRNA transcription levels. The fold activation or inhibition in all assays was measured relative to levels in non-differentiated empty-vector-transfected cells, set arbitrarily at 1 (horizontal black line or V). Data are shown as the mean ± SD of at least three biological assays performed in triplicate. The statistical significance of Student’s *t*-test results is indicated (* *p* ≤ 0.05, ** *p* ≤ 0.01, *** *p* ≤ 0.001). Non-statistical significance is indicated by ns.

Once overexpression of each individual NOTCH receptor was established, we performed luciferase assays to confirm whether each NOTCH receptor was activated and induced the transcription of NOTCH-receptor target genes (Figure 2).

Indeed, overexpression of each of the four NOTCH receptors resulted in the activation of the luciferase reporter gene in these pools, especially when *Notch1*, *Notch2*, and *Notch3* genes were overexpressed (Figure 2A). We also analyzed the effect of overexpressing *Notch* genes on NOTCH activation and signaling in C3H10T1/2 cells by measuring the relative mRNA transcription levels of the NOTCH-receptor target genes *Hes1* and *Hey1*. Stable overexpression of any NOTCH receptor increased *Hes1* gene expression. However, only overexpression of the NOTCH4 receptor activated *Hey1* gene expression. No significant changes in *Hey1* gene expression were observed in the stably NOTCH1-transfected pool and, unexpectedly, overexpression of NOTCH2 and NOTCH3 receptors diminished *Hey1* gene expression (Figure 2B).

The existence of different *Notch* genes and ligands, together with the controversial results obtained about their functions in cell differentiation processes, suggested to us that changes in the expression of one of these genes might exert an influence on the expression of the others. Thus, depending on the cellular context, a global change produced could be related to the final adipogenic potential and adipocyte fate. We recently revealed that such a feedback modulation among *Notch* and *Dlk* genes occurs in 3T3-L1 preadipocytes [18,19]. Here, we analyzed the probable existence of these feedback loops in C3H10T1/2 cells.

We observed that overexpression of any one of the *Notch* genes significantly affected the endogenous mRNA transcription levels of at least two other *Notch* genes in C3H10T1/2 cells (Figure 2C). Overexpression of the *Notch1* gene exerted no substantial effects on the expression of endogenous *Notch4* gene, but it increased the expression levels of endogenous *Notch2* and *Notch3* genes. Overexpression of the *Notch2* gene decreased the endogenous expression of all the other *Notch* genes. For its part, *Notch3* gene overexpression drastically upregulated the expression level of endogenous *Notch1* gene, decreased the expression of endogenous *Notch4* gene, and caused no significant change in endogenous *Notch2* gene expression. Finally, overexpression of *Notch4* gene upregulated endogenous *Notch3* gene expression, decreased endogenous *Notch1* gene expression, and caused no significant change in endogenous *Notch2* gene expression.

It is also known that NOTCH signaling can both positively and negatively regulate canonical and non-canonical ligand expression [89,90]. Recently, we observed that NOTCH receptor activity in 3T3-L1 preadipocytes modulates *Dlk* genes’ expression levels to different extents [8,17,18,19]. For these reasons, it was pertinent to analyze the effects of overexpression of each of the four NOTCH receptors on *Dlk* genes’ expression levels in C3H10T1/2 cells (Figure 2D). We found here that overexpression of *Notch1, Notch2,* and *Notch3* genes decreased endogenous *Dlk2* gene expression, but overexpression of *Notch4* gene increased it. However, overexpression of the *Notch1*, *Notch2,* or *Notch4* genes increased endogenous *Dlk1* gene expression in these cells, but *Notch3* gene overexpression downregulated it. One of the main target genes activated by NOTCH signaling is *Hes1*, the expression of which has been inversely related to *Dlk1* gene expression levels [18,19,35,91]. In this work, we pursued investigation of the effects of stable *Hes1* gene overexpression on *Dlk* genes’ expression in C3H10T1/2 cells (Figure 2E). As in 3T3-L1 cells [18,19], stable *Hes1* gene overexpression decreased *Dlk* genes’ expression.

On the other hand, we generated stable DLK1- and DLK2-transfected C3H10T1/2 pools and confirmed the overexpression of *Dlk1* and *Dlk2* mRNAs and proteins (Supplementary Figure S3A,B, respectively). Recently, we showed that both DLK proteins inhibit not only NOTCH1 receptor signaling [17,22,35], but also signaling from the other three NOTCH receptors in mouse cells [18,19]. Here, we confirmed using luciferase assays that stable or transient overexpression of both DLK1 and DLK2 proteins inhibited global NOTCH signaling in C3H10T1/2 cells (Supplementary Figure S3C,D, respectively). As positive controls for these luciferase assays, we determined NOTCH1 receptor signaling levels after transient overexpression of NICD1 (Supplementary Figure S3E) or the complete JAG1 canonical ligand (Supplementary Figure S3F). We also showed here that global NOTCH signaling was inhibited, as expected, by DAPT (Supplementary Figure S3G), at similar levels to those produced by overexpressing DLK proteins. In addition, as expected, stable overexpression of DLK proteins in C3H10T1/2 cells inhibited *Hes1* gene expression (Supplementary Figure S3H). However, overexpression of DLK2 protein activated *Hey1* gene expression, whereas no significant increase in *Hey1* gene expression was observed in cells overexpressing DLK1 protein (Supplementary Figure S3H).

We also observed that overexpression of DLK1 and DLK2 proteins affected the expression of *Notch* genes (Supplementary Figure S3I,J, respectively). Thus, overexpression of DLK1 protein downregulated the expression of the four *Notch* genes, whereas overexpression of DLK2 protein downregulated *Notch1* and *Notch3* genes expression, upregulated *Notch2* gene expression and caused no significant change in *Notch4* gene expression. Finally, we showed here that *Dlk1* gene overexpression upregulated *Dlk2* gene expression and vice versa in these cells (Supplementary Figure S3K,L, respectively).

All these data indicate the existence of a complex and coordinated feedback modulation process among *Notch* and *Dlk* genes that allows cells to respond adequately to external differentiation stimuli. Additionally, these changes must be dependent on the cellular context and may shape the outcome of adipogenesis and the phenotype of adipocytes.

### 3.3. Overexpression of NOTCH Receptors or DLK Proteins Enhances the Adipogenic Potential of Multipotent C3H10T1/2 Cells

As mentioned in the introduction, the role of NOTCH signaling in the adipogenesis process is highly controversial, with data indicating null, positive, or negative effects. These contradictory results could be due to the sensitivity of the cells to NOTCH activation levels, leading to different adipogenic outcomes in a concrete cellular context.

**Figure 3:**
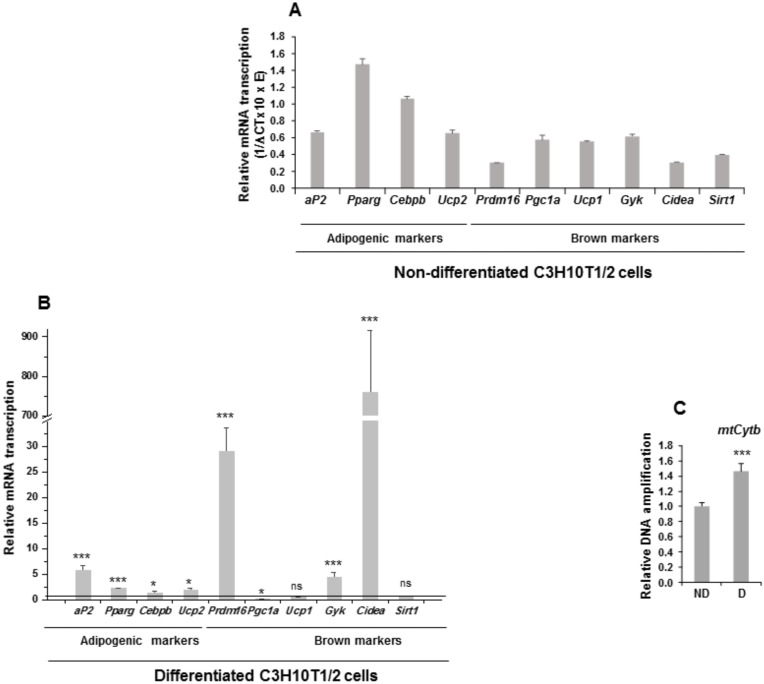
Expression of adipogenic markers and mitochondrial biogenesis markers in non-differentiated and differentiated C3H10T1/2 cells. (**A**) Relative mRNA transcription levels (1/∆ C_T_ (cycle threshold) × 10 × E (oligonucleotide efficiency)) of the adipogenic and brown adipocyte markers *aP2* (adipocyte protein 2/fatty acid binding protein 4), *Pparg* (peroxisome proliferator activated receptor gamma), *Cebpb* (CCAAT Enhancer Binding Protein Beta), *Ucp2* (uncoupling protein 2), *Prdm16* (PR domain-containing 16), *Pgc1a* (peroxisome proliferator activated receptor gamma coactivator 1-alpha), *Ucp1* (mitochondrial uncoupling protein 1)*, Gyk* (glycerol kinase), *Cidea* (cell death-inducing DNA fragmentation factor, alpha subunit-like effector A), and *Sirt1* (Sirtuin 1) in non-differentiated C3H10T1/2 cells. (**B**) Relative mRNA transcription levels of the same adipogenic and brown adipocyte markers in seven-day differentiated C3H10T1/2 cells. (**C**) qPCR analysis of mitochondrial *Cytb* DNA amplification (related to genomic *ApoB* (apoliprotein B) DNA amplification, see Materials and Methods section) in seven-day differentiated [D] and non-differentiated [ND] C3H10T1/2 cells. Data from qRT-PCR and qPCR assays were normalized to *P0* mRNA transcription levels. The fold activation or inhibition values were calculated relative to those of non-differentiated C3H10T1/2 cells, which were set arbitrarily at 1 (horizontal black line or ND). Data are shown as the mean ± SD of at least three biological assays performed in triplicate. The statistical significance calculated by Student’s *t*-test is indicated (* *p* ≤ 0.05, *** *p* ≤ 0.001). Non-statistical significance is indicated by ns.

We first analyzed the relative mRNA transcription levels of various adipogenic and mitochondrial biogenesis markers in non-differentiated and differentiated C3H10T1/2 cells (Figure 3).

We observed that all adipogenic markers were expressed in C3H10T1/2 cells (Figure 3A). When C3H10T1/2 cells were induced to differentiate to adipocytes, an increase in the expression of the adipogenic markers was produced, except for *Pgc1a*, for which expression decreased, and for *Ucp1* and *Sirt1*, which showed no significant changes in expression (Figure 3B). We also estimated the level of mitochondrial biogenesis by measuring the relative amount of *mtCytb* (mitochondrial cytochrome b) DNA in C3H10T1/2 cells. Thus, differentiated C3H10T1/2 cells showed higher relative *mtCytb* DNA levels than non-differentiated cells (Figure 3C). These results suggest that, in response to external adipogenesis signals, C3H10T1/2 cells may have acquired clear features proper of the mature brown adipocyte phenotype, as occurred in 3T3-L1 cells subjected to identical treatment [18,19].

**Figure 4:**
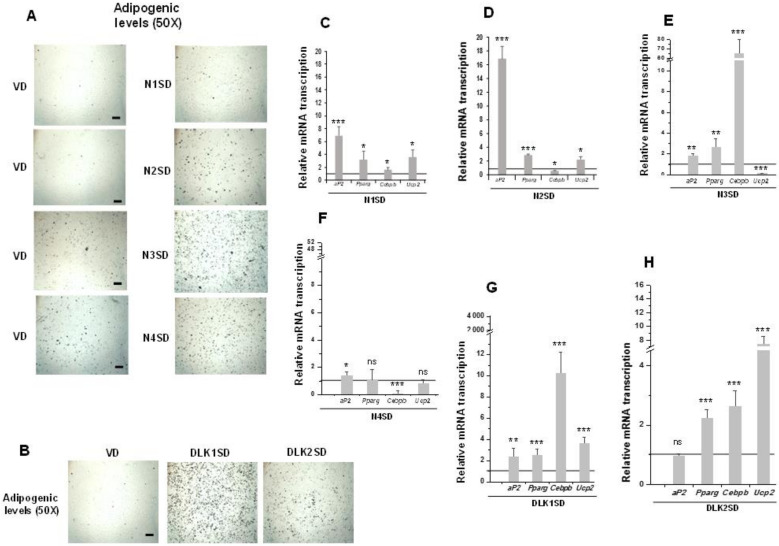
Stable overexpression of *Notch* and *Dlk* genes in multipotent C3H10T1/2 cells enhanced adipogenesis levels. Representative microscopic images of adipocytes from C3H10T1/2 cells transfected with *Notch* (**A**) or *Dlk* genes (**B**) showing their adipogenic levels (50× magnification images, scale bar 200 μm) after oil red O staining, all compared with the adipogenic levels of empty-vector transfected cells (VD). D: differentiated cells. qRT-PCR analysis of indicated adipogenic marker mRNA transcription levels in the differentiated stable *Notch1*-overexpressing cells (N1SD) (**C**), the differentiated stable *Notch2-*overexpressing cells (N2SD) (**D**), the differentiated stable *Notch3*-overexpressing cells (N3SD) (**E**), the differentiated stable *Notch4-*overexpressing cells (N4SD) (**F**), the differentiated stable *Dlk1*-overexpressing cells (DLK1SD) (**G**), and the differentiated stable *Dlk2*-overexpressing cells (DLK2SD) (**H**). Data from all qRT-PCR assays were normalized to *P0* mRNA transcription levels. The fold activation or inhibition levels in qRT-PCR assays were calculated relative to the levels shown by seven-day differentiated empty-vector-transfected cells, which were set arbitrarily at 1 (horizontal black line). Data are shown as the mean ± SD of at least three biological assays performed in triplicate. The statistical significance calculated by Student’s *t*-test is indicated (* *p* ≤ 0.05, ** *p* ≤ 0.01, *** *p* ≤ 0.001). Non-statistical significance is indicated by ns.

Upon observing these results, we decided to perform the same standard adipogenic assays to investigate the effects of overexpression of each of the four NOTCH receptors and DLK proteins on C3H10T1/2 adipogenesis levels (Figure 4).

Overexpression of NOTCH receptors, except for NOTCH4, clearly increased C3H10T1/2 adipogenesis to different levels, as determined by the increase in the number of adipocytes visualized by oil red O staining (Figure 4A, 50× magnification images) and by the increase in the expression of *aP2* and *Pparg* adipogenic markers (Figure 4C–F). *Cebpb* expression increased in *Notch1-* and *Notch3-* overexpressing cells, but it diminished in *Notch2-* and *Notch4-*overexpressing cells. Finally, the expression of *Ucp2* increased in differentiated *Notch1-* and *Notch2-*overexpressing cells, but it decreased in cells overexpressing *Notch3* gene and did not significantly change in *Notch4-*overexpressing cells.

We also confirmed in this work the increased adipogenic levels of C3H10T1/2 cells in the new transfected pools overexpressing DLK1 or DLK2 proteins, despite both DLK proteins being inhibitors of NOTCH signaling. Oil red O staining of differentiated cultures clearly revealed this increase (Figure 4B, 50× magnification images). Moreover, expression levels of the *Pparg*, *Cebpb,* and *Ucp2* markers increased in these differentiated *Dlk*-overexpressing cells (Figure 4G,H). However, *aP2* expression increased in differentiated *Dlk1*-overexpressing cells (Figure 4G), but it did not change significantly in *Dlk2*-overexpressing cells (Figure 4H).

It is known that the DLK1 protein can be processed at the membrane and its extracellular domain can be released to the external medium [92]. A previous work showed that the extracellular region of the DLK1 protein also enhances C3H10T1/2 cell adipogenesis [35], whereas it inhibits this process in 3T3-L1 preadipocytes [44,93,94,95]. Whether the DLK2 protein is also processed and its extracellular region released to the medium is unknown. Supplementary Figure S4A shows a Western blot analysis confirming the presence of DLK1, DLK2, JAG1, and DLL4 recombinant soluble proteins in HEK 293T/17 culture supernatants (see Section 2).

In this work, we performed standard adipogenesis assays with C3H10T1/2 cells by adding control conditioned culture media and conditioned culture media containing recombinant soluble DLK1 or DLK2 proteins (Supplementary Figure S4B). We confirmed that a recombinant soluble DLK1 protein enhanced, as expected, the adipogenesis of C3H10T1/2 cells, and we reveal for the first time that a recombinant soluble DLK2 protein was also able to enhance adipogenesis in these cells. We also analyzed the effect of the soluble canonical ligands JAG1 and DLL4 on C3H10T1/2 adipogenesis by adding control conditioned media or conditioned culture media containing each of those recombinant soluble canonical ligands (Supplementary Figure S4B). We observed that these canonical soluble ligands were also able to enhance the adipogenesis of C3H10T1/2 cells, compared with control media.

### 3.4. Overexpression of NOTCH3 Receptor or DLK Proteins Enhances the Brown Adipogenesis of Multipotent C3H10T1/2 Cells

**Figure 5:**
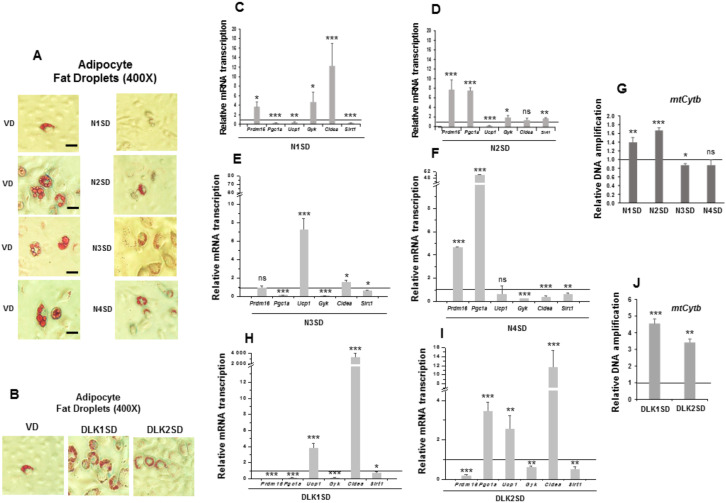
Stable overexpression of *Notch* and *Dlk genes* in multipotent C3H10T1/2 cells modulated brown adipogenesis. (**A**) Representative microscopic images of adipocytes of C3H10T1/2 cells transfected with *Notch* (**A**) or *Dlk* genes (**B**), showing the size of their lipid droplets (400× magnification images, scale bar 30 μm) after oil red O staining, all of them compared with the size of lipid droplets of empty-vector-transfected differentiated cells (VD). qRT-PCR analysis of the indicated brown marker expression levels in the differentiated stably *Notch1*-transfected cells (N1SD) (**C**), the differentiated stably *Notch2-*transfected cells (N2SD) (**D**), the differentiated stably *Notch3-*transfected cells (N3SD) (**E**), the differentiated stably *Notch4-*transfected cells (N4SD) (**F**), the differentiated stably *Dlk1*-transfected cells (DLK1SD) (**H**) and the differentiated stably *Dlk2*-transfected cells (DLK2SD) (**I**). (**G**) qPCR analysis of *mtCytb* (mitochondrial cytochrome b) DNA amplification (related to genomic *ApoB* DNA amplification; see Materials and Methods section) in seven-day differentiated C3H10T1/2 cells overexpressing *Notch* (**G**) and *Dlk* (**J**) genes. Data from qRT-PCR and qPCR assays were normalized to *P0* mRNA transcription levels. The fold activation or inhibition levels in PCR assays was calculated relative to the levels of seven-day differentiated empty-vector-transfected cells, which were set arbitrarily at 1 (horizontal black line). Data are shown as the mean ± SD of at least three biological assays performed in triplicate. The statistical significance calculated by Student’s *t*-test is indicated (* *p* ≤ 0.05, ** *p* ≤ 0.01, *** *p* ≤ 0.001). Non-statistical significance is indicated by ns.

Unlike 3T3-L1 preadipocytes, C3H10T1/2 cells are mesenchymal multipotent cells that can differentiate to adipocytes, myocytes, or osteoblasts, among other phenotypes, depending on extracellular stimuli. As described above, in response to the standard adipogenesis protocol, C3H10T1/2 cells generated mature adipocytes with small fat droplets and showed much lower adipogenesis levels than 3T3-L1 preadipocytes (Supplementary Figure S1A). We were interested in exploring whether overexpression of each of the four NOTCH receptors and DLK proteins could modify the pattern of expression of brown adipocyte and mitochondrial biogenesis markers and alter the size and/or number of cytoplasmic fat droplets (Figure 5).

We observed that 90% of C3H10T1/2 adipocytes overexpressing *Notch1*, *Notch2,* and *Notch3* genes exhibited a reduction in the size and an increase in the number of multilocular lipid droplets compared to their corresponding differentiated controls. The size and number of lipid droplets in C3H10T1/2 adipocytes overexpressing *Notch4* gene was similar to that of their controls (Figure 5A, 400× magnification). *Notch1* gene overexpression downregulated the expression of *Ucp1*, *Pgc1a*, and *Sirt1* in seven-day differentiated cells compared with controls, although it upregulated the expression of *Prdm16, Gyk*, and *Cidea* (Figure 5C). For its part, *Notch2* gene overexpression also downregulated *Ucp1* in seven-day differentiated cells, but the adipogenic induction upregulated the expression of *Prdm16*, *Pgc1a*, *Gyk,* and *Sirt1,* causing no significant effects in *Cidea* expression (Figure 5D). Interestingly, *Notch3* gene overexpression downregulated *Pgc1a, Gyk and Sirt1*, but it upregulated *Ucp1* and *Cidea* expression, and no significant changes were observed in *Prdm16* expression in seven-day differentiated cells (Figure 5E). Finally, *Notch4* gene overexpression upregulated *Prdm16* and *Pgc1a* expression, downregulated *Gyk, Cidea,* and *Sirt1* expression and caused no significant changes in *Ucp1* expression in seven-day differentiated cells (Figure 5F).

We also measured the relative amount of *mtCytb* DNA in these seven-day differentiated *Notch*-transfected pools, which indicates the grade of mitochondrial biogenesis. The relative amount of *mtCytb* DNA increased in differentiated *Notch1-* and *Notch2-*overexpressing cells, diminished in the differentiated stably *Notch3*-transfected pool, and did not change significantly in differentiated *Notch4*-overexpressing cells (Figure 5G). All these data suggest that overexpression of NOTCH receptors, except for NOTCH4, may enhance a brown-like phenotype in C3H10T1/2 adipocytes, especially in *Notch3*-overexpressing cells.

As modulators of NOTCH signaling, we expected that *Dlk1* or *Dlk2* genes overexpression would also affect the phenotype of mature adipocytes. We also observed that 90% of C3H10T1/2 adipocytes overexpressing *Dlk* genes exhibited a reduction in the size and an increase in the number of multilocular lipid droplets compared to their corresponding differentiated controls (Figure 5B, 400× magnification images). The expression of *Ucp1* and *Cidea* markers increased in the differentiated stably *Dlk1*-transfected pool, although the expression of *Prdm16*, *Pgc1a*, *Gyk* and *Sirt1* markers was downregulated (Figure 5H). In the case of the stably *Dlk2*-transfected pool, *Pgc1a*, *Ucp1,* and *Cidea* marker expression increased, and the expression of *Prdm16, Gyk* and *Sirt1* was downregulated in differentiated cells (Figure 5I). Finally, the relative amount of *mtCytb* DNA increased in both differentiated stably *Dlk*-transfected pools (Figure 5J). These results suggest that overexpression of DLK proteins in C3H10T1/2 cells could lead to a brown phenotype in adipocytes.

**Figure 6:**
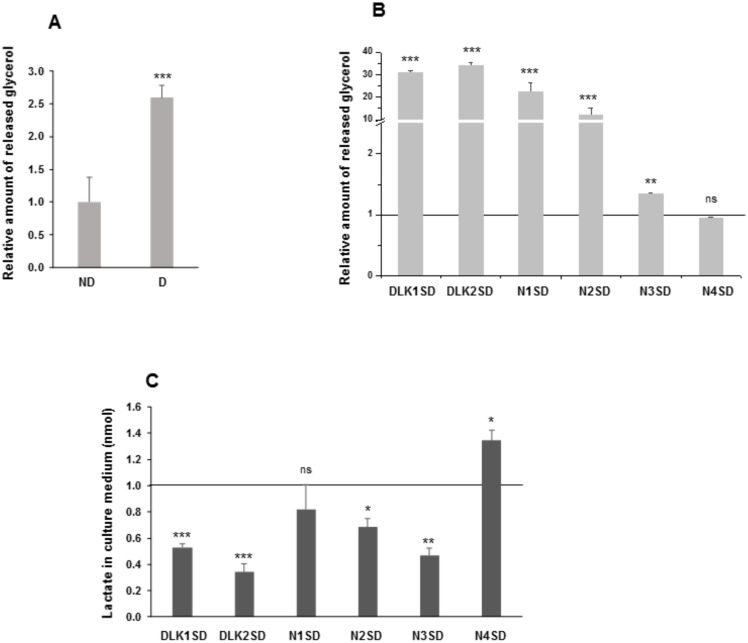
Lipolytic potential and release of lactate to the extracellular medium in C3H10T1/2 adipocytes overexpressing *Dlk* or *Notch* genes. (**A**) Relative levels of glycerol released to the extracellular medium in response to isoproterenol from non-differentiated (ND) and differentiated (D) C3H10T1/2 cells. (**B**) Relative levels of glycerol released to the extracellular medium in response to isoproterenol from differentiated *Dlk-* or *Notch-*overexpressing cells. (**C**) Relative levels of lactate released into the culture media of differentiated *Dlk-* or *Notch-*overexpressing C3H10T1/2 cells. The fold activation or inhibition levels were calculated relative to the levels of non-differentiated cells (**A**) or empty-vector-differentiated cells (**B**,**C**), which were set arbitrarily at 1 (horizontal black line or ND cells). Data are shown as the mean ± SD of at least three biological assays performed in triplicate. The statistical significance calculated by Student’s *t*-test is indicated (* *p* ≤ 0.05, ** *p* ≤ 0.01, *** *p* ≤ 0.001). Non-statistical significance is indicated by ns.

To further analyze the effects of overexpression of *Notch* and *Dlk* genes on mature adipocyte fate, we measured the amount of glycerol released to the medium by inducing lipolysis with the β-adrenergic agonist isoproterenol, and the amount of extracellular lactate (Figure 6).

Differentiated C3H10T1/2 cells (Figure 6A) and differentiated C3H10T1/2 transfected pools overexpressing the *Notch1*, *Notch2, Notch3*, *Dlk1,* or *Dlk2* genes (Figure 6B) showed higher levels of glycerol release compared with their respective non-differentiated cells or empty-vector-differentiated cells, respectively. However, no significant differences were observed in differentiated cells overexpressing *Notch4* gene, compared with its control. We also analyzed the amount of lactate released to the extracellular medium, which indirectly provides an extracellular acidification rate (ECAR) (Figure 6C). Compared to their corresponding controls, differentiated C3H10T1/2 transfected pools overexpressing *Dlk1*, *Dlk2*, *Notch2,* or *Notch3* genes showed lower levels of lactate released to the extracellular medium, whereas the differentiated transfected pool overexpressing *Notch4* gene released higher levels of lactate compared with its control. No significant differences were observed in cells overexpressing *Notch1* gene.

**Figure 7:**
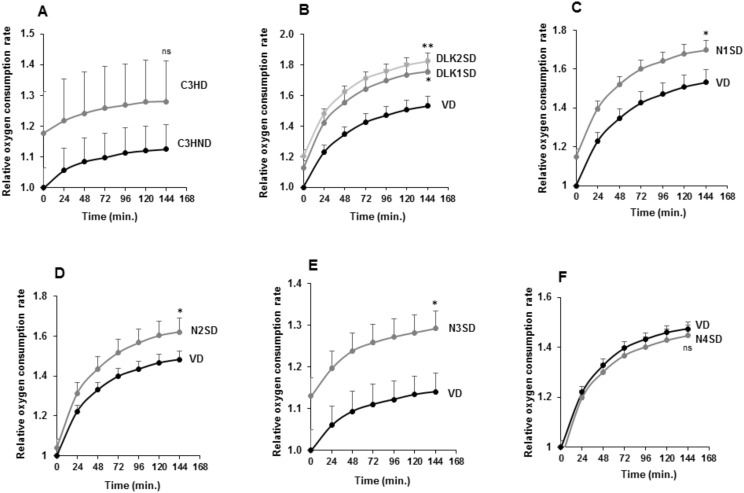
Oxygen consumption rate (OCR) in C3H10T1/2 adipocytes overexpressing *Dlk* or *Notch* genes. (**A**) Relative oxygen consumption rate (OCR) in non-differentiated (C3HND) and differentiated (C3HD) C3H10T1/2 cells. Relative oxygen consumption rate (OCR) in differentiated C3H10T1/2 cells overexpressing *Dlk* (**B**), *Notch1* (**C**), *Notch2* (**D**), *Notch3* (**E**), or *Notch4* (**F**) genes. The fold activation or inhibition levels were calculated relative to time 0 of non-differentiated cells, and, additionally, to OCR of non-differentiated cells (ND) (**A**) or seven-day differentiated empty-vector-transfected cells (VD) (**B**–**F**), which was set arbitrarily at 1. Data are shown as the mean ± SD of at least three biological assays performed in triplicate. The statistical significance calculated by Student’s *t*-test is indicated at 144 min (* *p* ≤ 0.05, ** *p* ≤ 0.01). Non-statistical significance is indicated by ns.

Finally, we analyzed the oxygen consumption rate (OCR), which is a measure of cellular respiration and mitochondrial function, using a phosphorescent oxygen probe (Figure 7).

As shown, the increased OCR in differentiated C3H10T1/2 cells was not significant (Figure 7A). However, we observed higher OCR levels in differentiated transfected pools overexpressing the *Dlk1*, *Dlk2*, *Notch1*, *Notch2,* or *Notch3* genes when compared with their respective differentiated controls (Figure 7B–E). On the other hand, the OCR change in differentiated C3H10T1/2 cells overexpressing *Notch4* gene was also not significant (Figure 7F).

## 4. Discussion

The inhibitory role of DLK1 and DLK2 proteins on NOTCH activation and signaling [8,17,18,19,20,21,22,35] has been confirmed in many works of the scientific community [4,17,22,23,32,35,37,96,97,98,99,100,101,102]. Recently, we observed that both DLK proteins inhibit each of the four NOTCH receptors’ activities [18,19]. However, some authors have also reported that the DLK1 protein does not affect NOTCH signaling [37,103,104,105,106], or even that it activates NOTCH signaling [25,91,107,108,109]. Together with the existence of different DLK1 protein and probably DLK2 protein isoforms, self- and cross-interactions previously reported [22,110,111] could explain some of the contradictory effects reported for DLK proteins on NOTCH activation and signaling.

Many conflicting results have arisen about the role of NOTCH signaling in the adipogenesis process [18,19,28,29,30,31,32,33,34,35,112,113,114]. Recently, we showed that overexpression of each of the four NOTCH receptors in 3T3-L1 preadipocytes enhances their adipogenic levels, and that overexpression of DLK proteins inhibits, as expected, this differentiation process in these cells [18,19]. Interestingly, we also showed that, contrary to inhibiting, overexpression of *Dlk1* gene or downregulation of *Dlk2* gene enhanced the adipogenic process in C3H10T1/2 cells [35].

In this work, we used multipotent C3H10T1/2 cells as an adipogenic model. We analyzed the effects of NOTCH receptors and we confirmed the effect of both DLK proteins on the adipogenesis process of C3H10T1/2 cells, which are able to differentiate to adipocytes, but also to myocytes or bone cells depending on the extracellular signals provided. To understand the different effects of DLK proteins on 3T3-L1 and C3H10T1/2 adipogenesis, we first compared the expression patterns of *Notch* family genes between both cell lines. We observed that the expression levels of *Notch* genes and of their target genes *Hes1* and *Hey1* were higher in C3H10T1/2 than in 3T3-L1 cells, but *Dlk* genes’ expression levels were higher in 3T3-L1 cells than in C3H10T1/2 cells. These results are consistent with the higher global NOTCH receptor activity previously observed in C3H10T1/2 cells compared to 3T3-L1 preadipocytes [35], which could explain the lower adipogenic levels of these cells compared with 3T3-L1 preadipocytes.

We observed that *Notch2* and *Notch3* genes’ overexpression, unexpectedly, led to lower *Hey1* expression. This result could be explained by the existence of a specific and selective activation of NOTCH-receptor targets depending on the NOTCH receptor and/or canonical ligand involved, as well as by the effects of *Notch* genes’ overexpression on the expression levels of the other NOTCH receptors and ligands. Ong and coworkers performed parallel assays of three different *Notch*-gene-responsive promoters in several cell lines and revealed that relative activation strength was dependent on the protein module and promoter context [115]. On the other hand, Beatus and coworkers showed that the NICD1 protein is a potent activator of *Hes* gene promoters, whereas NICD3 is a much weaker activator and can even repress the activation of NICD1-mediated *Hes* gene promoters in certain contexts [116,117]. The unexpected activation of *Hey1* expression in *Dlk2*-overexpressing cells could also have been due to a specific and selective action of DLK proteins on each of the four NOTCH receptors. Stable *Hes1* gene overexpression, which mimics a continuous activation of NOTCH signaling, decreased endogenous *Dlk* genes’ expression, in contrast to what occurred when *Notch* genes were overexpressed. The different effects of overexpression of each of the four *Notch* genes on the other *Notch* genes’ expression and the effects that other NOTCH targets besides HES1 could have on *Dlk* genes´ expression could possibly help to explain these results.

Even though *Notch1* gene expression is upregulated at the end of the adipogenic process, the expression levels of the rest of *Notch* genes and the NOTCH-receptor target genes *Hes1* and *Hey1* were downregulated. These results suggest that NOTCH1 receptor may be required throughout the entire adipogenic process, whereas the rest of the NOTCH receptors and DLK proteins may be only necessary at the beginning of adipogenesis.

It has been reported that DLK1 protein is processed by the protease TACE (ADAM metallopeptidase domain 17) [92], and that the soluble form can inhibit adipogenesis in 3T3-L1 cells but activate it in C3H10T1/2 cells. Whether DLK2 protein is similarly processed to generate a secreted soluble form is not known. We report here that conditioned media containing recombinant DLK2 soluble protein added over the entire adipogenic process also enhanced C3H10T1/2 adipogenesis. On the other hand, it has been reported that all membrane-bound NOTCH receptors’ canonical ligands activate NOTCH receptors. However, these ligands can also be processed, and some authors have revealed that their soluble forms can inhibit NOTCH signaling [118,119]. Thus, when we added the soluble recombinant canonical ligands JAG1 and DLL4 to the adipogenic cocktail, we observed that they also enhanced adipogenesis in C3H10T1/2 cells, which suggests that these two soluble canonical ligands may behave as the soluble DLK proteins do by acting as inhibitors of NOTCH signaling in these cells.

Taking into account the above results, it could be expected that an increase in NOTCH receptors’ expression, activation, and signaling in C3H10T1/2 cells should result in a decrease in their adipogenesis levels. This fact would be also in agreement with the fact that the overexpression of DLK proteins or their soluble forms activates adipogenesis via their action as inhibitors of NOTCH signaling. Unexpectedly, overexpressing NOTCH receptors, except for NOTCH4, in C3H10T1/2 cells clearly activated the adipogenesis process to different degrees, as indicated by adipogenic markers. We do not know why an increase in the NOTCH1, -2, and -3 receptors’ activities in C3H10T1/2 cells bypassed the apparent negative overall effect of NOTCH signaling on adipogenesis in these cells. We can speculate that a fine regulation is needed to generate particular and global NOTCH signaling levels, which could permit adipogenesis to proceed or not in response to extracellular signals. These particular NOTCH signaling levels could be highly dependent on the cellular context or the feedback modulation among *Notch* and *Dlk* genes we have observed,

It is also possible that the overexpression of NOTCH receptors in multipotent C3H10T1/2 cells could actually enhance a brown adipogenesis process instead of a typical white adipogenesis process. In relation to this possibility, we analyzed the expression levels of brown adipocyte and mitochondrial biogenesis markers in transfected cell pools overexpressing each of the four NOTCH receptors or DLK proteins. Only *Notch3-*overexpressing cells showed a high increase in *Ucp1* expression, indicative of a brown-like phenotype. The other three *Notch-*overexpressing cells showed an increase in some brown mitochondrial biogenesis markers, which would modulate the expression of *Ucp1* and other factors to produce a final brown-like phenotype. On the other hand, overexpression of any of the DLK proteins also increased *Ucp1* expression, indicating that a brown-like adipocyte phenotype was acquired by differentiated C3H10T1/2 cells. In summary, we observed that overexpression of *Notch3* or *Dlk* genes exerted the greatest effects on the expression of brown and mitochondrial biogenesis markers. These data are also in agreement with the reduction in size and increase in the number of multilocular lipid droplets by these adipocytes, compared with their corresponding differentiated controls, *Notch4* gene overexpression being the one exerting minimal or no effects.

To further confirm the effects of *Notch* and *Dlk* genes’ overexpression on mature adipocyte fate, we measured the lipolytic potential, the release of lactate to the extracellular medium, and the oxygen consumption rate of C3H10T1/2 cells overexpressing these genes. Except for *Notch4*, overexpression of *Notch* and *Dlk* genes led to a higher lipolytic potential, a higher rate of oxygen consumption, and a decrease in the release of extracellular lactate, a molecule that can be used, together with fatty acids, as an energy source by brown adipose tissue, among others.

The results presented in this work indicate that overexpression of NOTCH1, -2, and 3 receptors or DLK proteins modifies the expression of brown and mitochondrial biogenesis markers and the metabolic features of adipocytes. Thus, NOTCH1, -2, and -3 receptors’ or DLK proteins’ overexpression may drive the differentiation of C3H10T1/2 cells towards different types of adipocytes in response to extracellular signals, producing features of brown-like adipocytes. The conversion of C3H10T1/2 cells to a brown-like adipocyte phenotype was more intense in the case of the overexpression of any DLK protein or the NOTCH3 receptor, despite the fact that these cells show reduced levels of mitochondrial biogenesis markers and a lower lipolytic potential.

Our data suggest that there may be a decrease or an increase in the global NOTCH signaling levels depending on the differential expression of these genes, the feedback modulation among them, and the stoichiometry of the interactions and affinities among these proteins. These changes in NOTCH activation and signaling may potentiate or inhibit adipogenesis and brown–white adipocyte fate conversion in response to extracellular stimuli and depending on the cell context. Consistent with our findings, it has been reported that different levels of NOTCH activation and signaling, including the level of active NOTCH1 receptor, determines distinct cellular responses in several processes [20,120,121]. Our data show that low and high levels of NOTCH activation and high levels of NOTCH activation may be favorable to brown adipogenesis. It has been described that oscillatory *Hes1* gene expression could be a feature in several cellular events [122,123,124]. Our results suggest for the first time that the level of NOTCH signaling plays an important role in adipogenesis and adipocyte fate.

**Figure 8:**
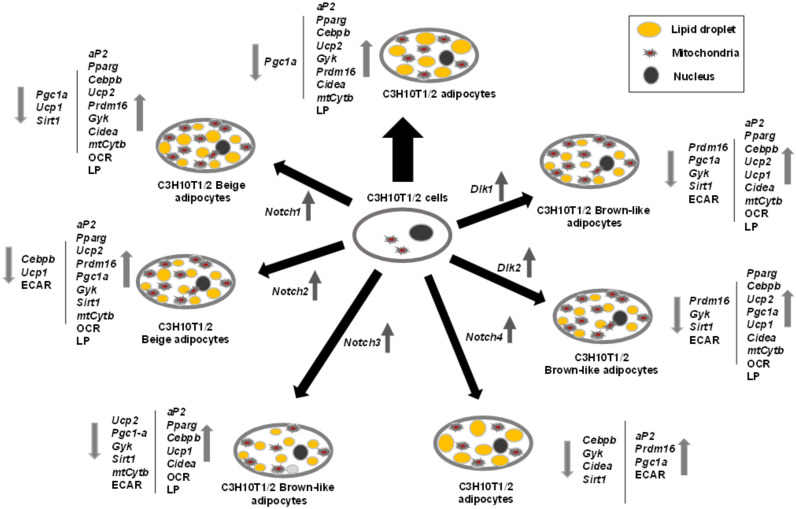
Outline of the roles of *Notch* and *Dlk* genes’ overexpression on C3H10T1/2 adipogenesis. The effects of adipogenic inductors at the end of the differentiation process (2 days with dexamethasone and IBMX (3-Isobutyl-1-methylxanthine) and 5 days with insulin) on C3H10T1/2 cells (wide black arrow) are compared to the effects of the same adipogenic inductors on C3H10T1/2 cells stably overexpressing each of the *Notch* or *Dlk* genes. The direction (up or down) of the variations in the expression levels of adipogenic markers, brown adipocyte markers, mitochondrial biogenesis markers, and metabolic effects are shown by grey arrows. OCR: oxygen consumption rate, ECAR: extracellular acidification rate, LP: lipolytic potential.

Figure 8 summarizes the effects of NOTCH receptors and DLK proteins on C3H10T1/2 adipogenesis and adipocyte browning revealed in this work.

Some authors have already pointed to the browning of adipose tissue as a promising therapeutic approach for obesity [125,126], since converting white to brown adipocytes would lead to burning the fat accumulated in the cells. The modulation of NOTCH signaling aimed at inhibiting the generation of white fat cells and stimulating the generation of brown adipocytes may constitute a promising strategy to regulate fat mass in humans and fight against obesity and type 2 diabetes.

## 5. Conclusions

These are the main conclusions of this work:

1. C3H10T1/2 mesenchymal cells display lower DLK expression levels and higher NOTCH expression, activity, and signaling levels than 3T3-L1 preadipocytes.
2. There are complex feedback regulation loops among *Notch* and *Dlk* genes in C3H10T1/2 cells.
3. Even though *Notch1* gene expression is upregulated at the end of the adipogenic process, the expression of the rest of *Notch* genes and NOTCH receptors’ target genes *Hes1* and *Hey1* is downregulated.
4. The overexpression of each of the four NOTCH receptors, except for NOTCH4, enhances the adipogenic levels of multipotent C3H10T1/2 cells.
5. NOTCH activation levels can modulate the adipocyte fate of multipotent C3H10T1/2 cells.
6. The overexpression of NOTCH3 receptor or DLK proteins determine a brown adipogenesis fate in multipotent C3H10T1/2 adipocytes.

## Figures and Tables

**Table 1:** Primary and secondary antibodies used in Western blot analysis.

Protein	Dilution of Primary and Secondary Antibodies	Company
NOTCH1	Rabbit anti-NOTCH1 C20R (1:1000)	Santa Cruz Biotechnology
NOTCH2	Goat anti-NOTCH2 M20 (1:500)	Santa Cruz Biotechnology
NOTCH3	Rabbit anti-NOTCH3 (1:1000)	Abcam
NOTCH4	Rabbit anti-NOTCH4 (1:1000)	Upstate Millipore
HA	Mouse anti-HA 16B12 (1:5000)	Covance
DLK1 (DELTA-like 1 homolog)	Rabbit anti-DLK1 (1:1000)	Nueda et al., 2008
DLK2 (DELTA-like 2 homolog)	Rabbit polyclonal anti-mouse DLK2-C-terminal (1:500)	Abcam
α-Tubulin	Mouse anti-alpha-Tubulin (1:5000)	Sigma

## Supplementary Materials

The following are available online at https://www.mdpi.com/2073-4409/9/9/2032/s1. Figure S1: *Comparison of adipogenesis levels and expression levels of some of the Notch family genes between multipotent C3H10T1/2 cells and 3T3-L1 preadipocytes*. Figure S2: Stable overexpression of NOTCH receptors in mesenchymal C3H10T1/2 cells. Figure S3: Overexpression of DLK proteins and NOTCH activation and signaling levels in mesenchymal C3H10T1/2 cells stably-overexpressing DLK proteins. Figure S4: Effect of recombinant soluble canonical and non-canonical ligands of NOTCH receptors on the adipogenesis process of mesenchymal C3H10T1/2 cells.

## Abbreviations

**aP2**	adipocyte protein 2/fatty acid binding protein 4.
**APOB**	apoliprotein B.
**BAT**	brown adipose tissue.
**CEBPβ**	CCAAT Enhancer Binding Protein Beta.
**CIDEA**	cell death-inducing DNA fragmentation factor, alpha subunit-like effector A.
**CM**	conditioned medium.
**CSL/RBPJκ**	CBF1, Suppressor of Hairless, Lag-1/recombination signal binding protein for immunoglobulin kappa J region.
**C_T_**	cycle threshold.
**DAPT**	N-[N-(3, 5-Difluorophenacetyl)-L-alanyl]-S-phenylglycine t-butyl ester.
**DLK1**	DELTA-like 1 homolog.
**DLK2**	DELTA-like 2 homolog.
**DLK**	DELTA-like homolog.
**DLL4**	DELTA-Like Canonical NOTCH Ligand 4.
**DNER**	DELTA/NOTCH EGF-like Repeat Containing.
**DOS**	DELTA and OSM-11 domain.
**DSL**	DELTA-SERRATE-LAG-2.
**E**	oligonucleotide efficiency.
**ECAR**	extracellular acidification rate.
**EGFL7**	Epidermal growth factor-like protein 7.
**GYK**	*g*lycerol kinase.
**HA**	influenza hemagglutinin.
**HES1**	hairy and enhancer of split 1.
**HEY1**	hairy and enhancer-of-split related with YRPW motif 1.
**IBMX**	3-Isobutyl-1-methylxanthine.
**JAG1**	JAGGED canonical NOTCH ligand 1.
**LP**	lipolytic potential.
**MSCs**	mesenchymal stem cells.
**mtCYTB**	mitochondrial cytochrome b.
**NICD**	intracellular active domain of NOTCH receptors.
**OCR**	oxygen consumption rate.
**P0**	ribosomal protein P0
**PGC1α**	peroxisome proliferator activated receptor gamma coactivator 1-alpha.
**PPARγ**	peroxisome proliferator activated receptor gamma.
**PRDM16**	PR domain-containing 16.
**qPCR/qRT-PCR**	quantitative-polymerase chain reaction/quantitative reverse transcription-polymerase chain reaction.
**SIRT1**	Sirtuin 1.
**TACE**	ADAM metallopeptidase domain 17.
**UCP1**	mitochondrial uncoupling protein 1.
**UCP2**	uncoupling protein 2.
**WAT**	white adipose tissue.
