# Supplementary material for: NOTCH Receptors and DLK Proteins Enhance Brown Adipogenesis in Mesenchymal C3H10T1/2 Cells
Source: Cells. 2020 Sep 4;9(9):2032. doi: 10.3390/cells9092032 (PMC7565505; doi:10.3390/cells9092032)
Supplement: Supplementary file 1 [file cells-09-02032-s001.pdf]

SUPPLEMENTARY DATA

NOTCH RECEPTORS AND DLK PROTEINS ENHANCE BROWN ADIPOGENESIS IN MESENCHYMAL C3H10T1/2 CELLS

María-Milagros Rodríguez-Cano, María-Julia González-Gómez, Beatriz Sánchez-Solana, Eva María Monsalve, María-José M. Díaz-Guerra, Jorge Laborda, María Luisa Nueda, and Victoriano Baladrón.

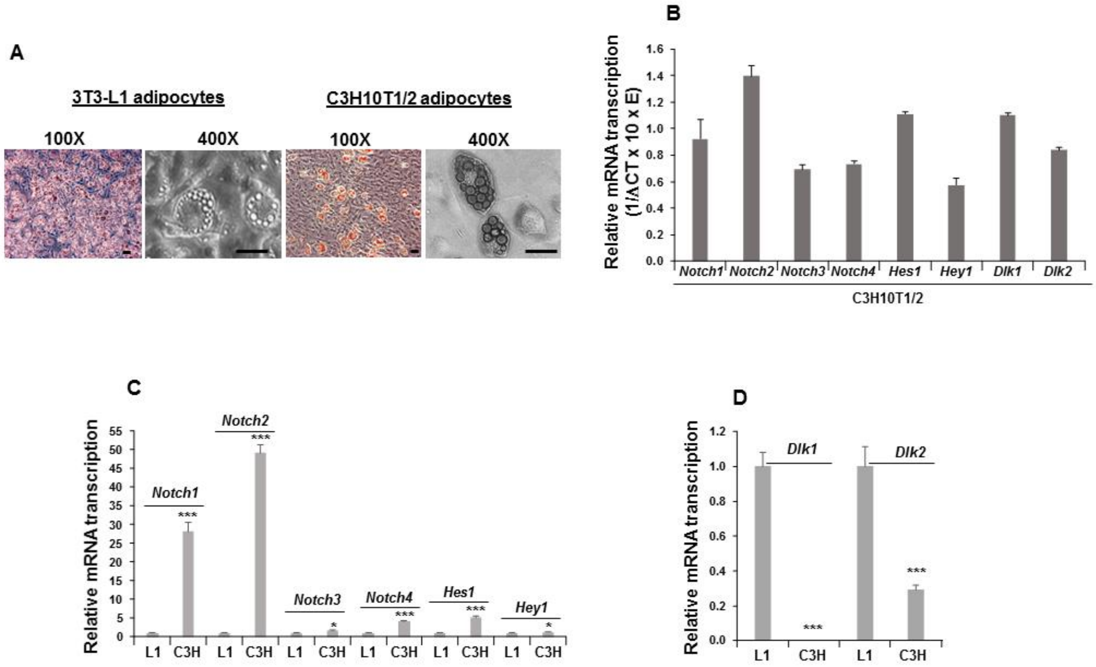

Figure S1. ***Comparison of adipogenesis levels and expression levels of some of the Notch family genes between multipotent C3H10T1/2 cells and 3T3-L1 preadipocytes.*** (A) Representative microscopy images of non-transfected C3H10T1/2 and 3T3-L1 adipocytes seven days after standard adipogenic induction and oil red O staining (see Material and Methods). Adipogenic levels (100x magnification, scale bar 100 μm) and fat droplet size (400x magnification, scale bar 30 μm) are shown. (B) Relative mRNA transcription levels (1/Δ C_T (cycle threshold) x 10 x E [Oligonucleotide efficiency]) of *Notch, Dlk, Hes1* and *Hey1* genes in non-differentiated C3H10T1/2 cells. (C) Comparison of *Notch, Hes1* and *Hey1* mRNA transcription levels between non-differentiated 3T3-L1 (L1) and C3H10T1/2 (C3H) cells. (D) Comparison of *Dlk1* and *Dlk2* mRNA transcription levels between non-differentiated 3T3-L1 (L1) and C3H10T1/2 (C3H) cells. Data in all qRT-PCR assays were normalized to *P0* mRNA transcription levels. The fold activation in assays C and D panels is calculated relative to the levels of the non-differentiated 3T3-L1 (L1) cells, set arbitrarily at 1. Data are shown as the mean ± SD of at least three biological replicates. The statistical significance of Student's *t*-tests results is indicated (* $p \leq 0.05$, *** $p \leq 0.001$).

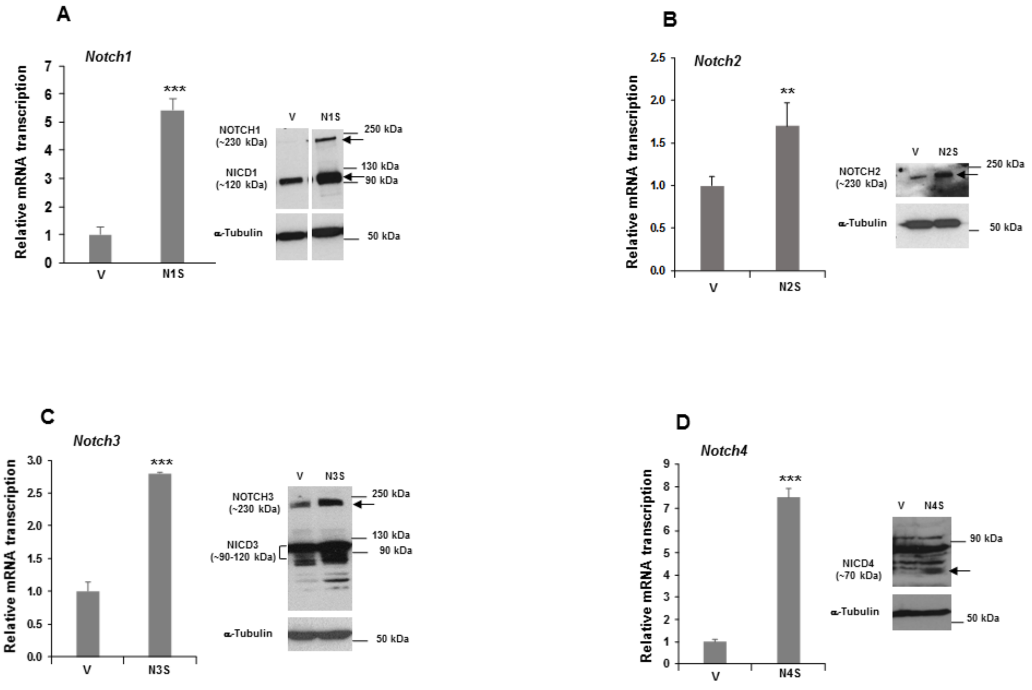

Figure S2. Stable overexpression of NOTCH receptors in mesenchymal C3H10T1/2 cells. qRT-PCR analysis of the relative mRNA transcription levels (left panel) and representative Western blots indicative of protein levels (right panel) in the stable *Notch1*-overexpressing cells (N1S) (A), the stable *Notch2*-overexpressing cells (N2S) (B), the stable *Notch3*-overexpressing cells (N3S) (C), and the stable *Notch4*-overexpressing cells (N4S) (D) used in this work. In the case of *Notch1*-overexpressing cells, the intracellular NOTCH1 (NICD1) protein and the complete NOTCH1 protein levels are shown. In *Notch2*-overexpressing cells, the complete protein level is shown. In the case of *Notch3*-overexpressing cells, the intracellular NOTCH3 (NICD3) protein and complete NOTCH3 protein levels are shown. In *Notch4*-overexpressing cells, the intracellular NOTCH4 (NICD4) protein level is shown. The expression of alpha-tubulin was used as a loading and quality control in all Western blots. Data in all qRT-PCR assays were normalized to *P0* mRNA transcription levels. The fold activation in qRT-PCR assays was calculated relative to the levels of non-differentiated empty-vector-transfected cells, set arbitrarily at 1 (V). Data are shown as the mean ± SD of at least three biological replicates. The statistical significance of Student's t-tests results is indicated (** $p \leq 0.01$, *** $p \leq 0.001$).

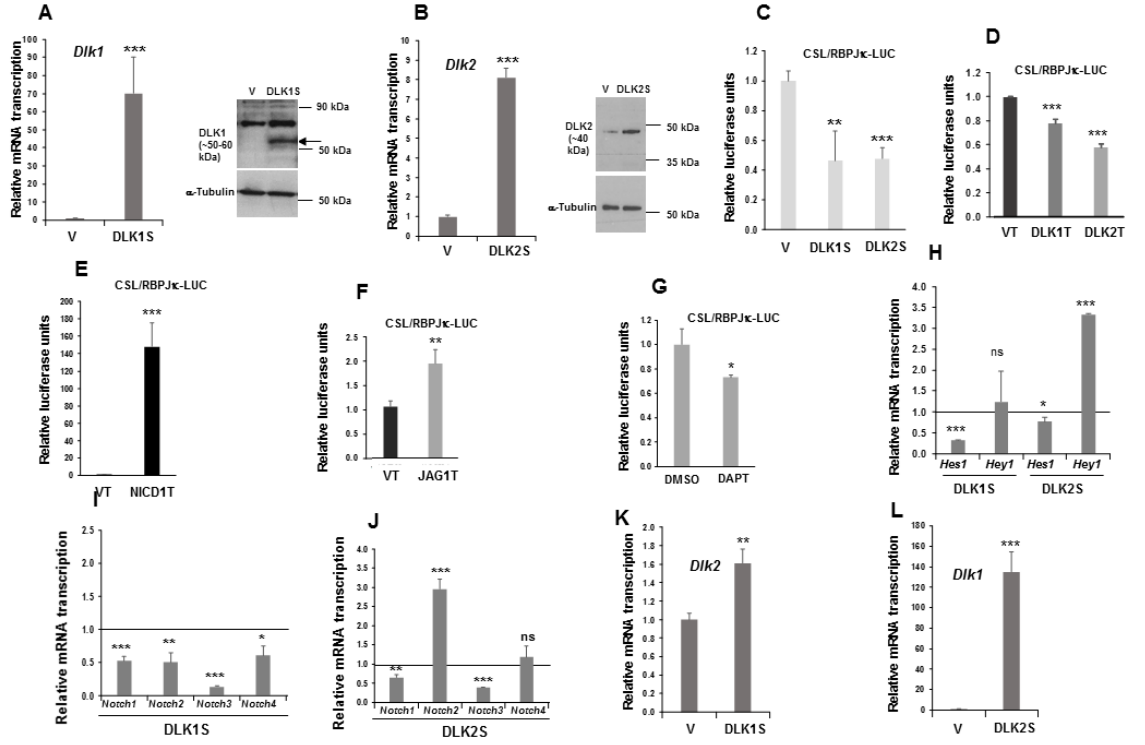

Figure S3. **Overexpression of DLK proteins and NOTCH activation and signaling levels in mesenchymal C3H10T1/2 cells stably-overexpressing DLK proteins.** qRT-PCR analysis of the relative mRNA transcription levels (left panel) and representative Western blot of protein levels (right panel) in non-differentiated stable *Dlk1*-overexpressing cells (DLK1S) (A) and stable *Dlk2*-overexpressing cells (DLK2S) (B). The expression of alpha-tubulin was used as a loading and quality control in Western blots. NOTCH transcriptional activity (CSL/RBPJk-LUC), as measured by luciferase reporter gene assays, in reference to control cells, in each of the two *Dlk*-stably- (DLK1S, DLK2S) (C) or transiently- (DLK1T, DLK2T) (D) transfected cells. NOTCH transcriptional activity (CSL/RBPJk-LUC), as measured by luciferase reporter gene assays, in C3H10T1/2 cells transiently-transfected with the NICD1-expression plasmid (E), the complete JAGGED1-expressing plasmid (F), and in C3H10T1/2 cells treated or not with DAPT (G), used as positive or negative controls. The relative luciferase activities were always normalized with renilla values. (H) qRT-PCR analysis of the relative *Hes1* and *Hey1* mRNA transcription levels in each of the stable *Dlk*-overexpressing cells. qRT-PCR analysis of the relative *Notch* (I and J) and *Dlk* (K and L) mRNA transcription levels in the stable *Dlk1*-overexpressing cells (I and K) and the stable *Dlk2*-overexpressing cells (J and L). In all qRT-PCR assays, data were normalized to *P0* mRNA transcription levels. The fold activation or inhibition in all assays was measured relative to the levels of control cells in each case, set arbitrarily at 1 (V [stably-empty-vector-transfected cells] [C]; VT [transiently-empty-vector-transfected cells] [D, E and F]; DMSO-treated cells [G]; and horizontal black line [stably-empty-vector-transfected cells] [H, I and J]). Data are shown as the mean ± SD of at least three biological assays performed in triplicate. The statistical significance of Student's *t*-test results is indicated (* p ≤ 0.05, ** p ≤ 0.01, *** p ≤ 0.001). Non-statistical significance is indicated by ns.

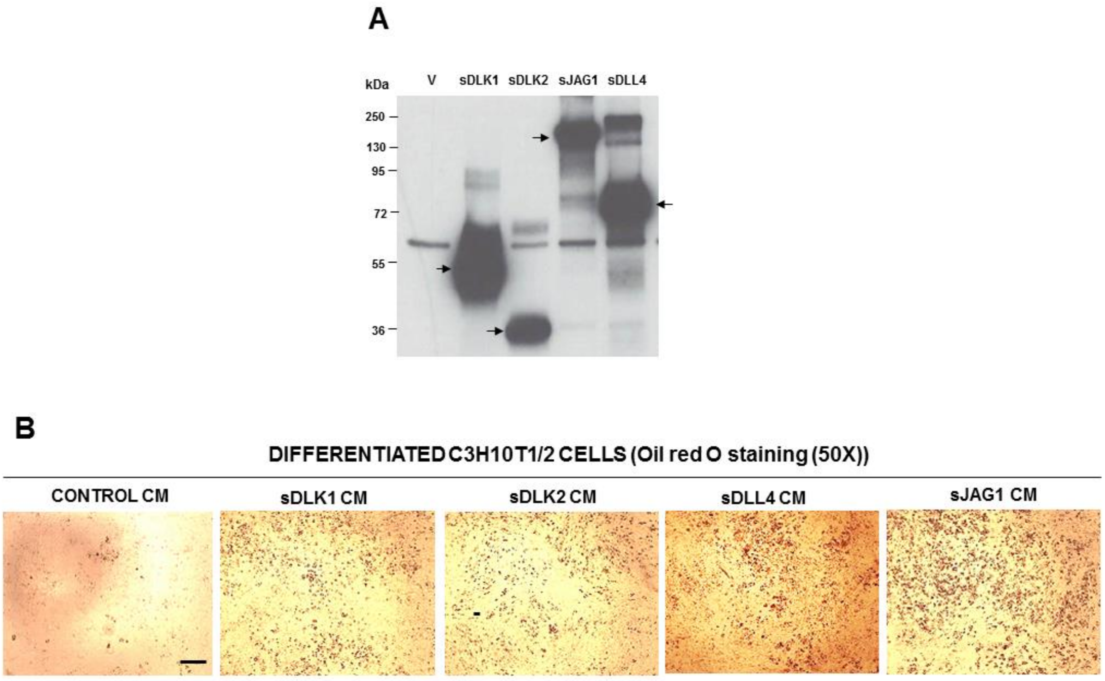

Figure S4. Effect of recombinant soluble canonical and non-canonical ligands of NOTCH receptors on the adipogenesis process of mesenchymal C3H10T1/2 cells. (A) Representative Western blot (Mouse monoclonal anti-HA 16B12 primary antibody) showing the expression and secretion of sDLK1, sDLK2, sJAG1 or sDLL4 soluble ligands of NOTCH receptors to the culture medium of HEK 293T/17 cells. V: control supernatant of HEK 293T/17 cells transfected with the empty-vector. (B) Representative microscopy images visualized by oil red O staining (50x magnification images, scale bar 200 µm). The images correspond to C3H10T1/2 adipocytes seven days after standard adipogenic induction in the presence, during the entire adipogenic treatment, of control conditioned medium (CONTROL CM) or conditioned medium containing a soluble ligand of NOTCH receptors (sDLK1 CM, sDLK2 CM, sJAG1 CM or sDLL4 CM).
